# Magnetotactic Bacteria from Extreme Environments

**DOI:** 10.3390/life3020295

**Published:** 2013-03-26

**Authors:** Dennis A. Bazylinski, Christopher T. Lefèvre

**Affiliations:** 1 University of Nevada at Las Vegas, School of Life Sciences, Las Vegas, Nevada, 89154-4004, USA; 2 CEA Cadarache/CNRS/Aix-Marseille Université, UMR7265 Service de Biologie Végétale et de Microbiologie Environnementale, Laboratoire de Bioénergétique Cellulaire, 13108, Saint-Paul-lez-Durance, France; E-Mail: lefevrechristopher@hotmail.com

**Keywords:** magnetotactic bacteria, biomineralization, magnetite, greigite, biodiversity and ecology, extreme environments, extremophiles, astrobiology

## Abstract

Magnetotactic bacteria (MTB) represent a diverse collection of motile prokaryotes that biomineralize intracellular, membrane-bounded, tens-of-nanometer-sized crystals of a magnetic mineral called magnetosomes. Magnetosome minerals consist of either magnetite (Fe_3_O_4_) or greigite (Fe_3_S_4_) and cause cells to align along the Earth’s geomagnetic field lines as they swim, a trait called magnetotaxis. MTB are known to mainly inhabit the oxic–anoxic interface (OAI) in water columns or sediments of aquatic habitats and it is currently thought that magnetosomes function as a means of making chemotaxis more efficient in locating and maintaining an optimal position for growth and survival at the OAI. Known cultured and uncultured MTB are phylogenetically associated with the *Alpha*-, *Gamma*- and *Deltaproteobacteria* classes of the phylum *Proteobacteria*, the *Nitrospirae* phylum and the candidate division OP3, part of the *Planctomycetes*-*Verrucomicrobia*-*Chlamydiae* (PVC) bacterial superphylum. MTB are generally thought to be ubiquitous in aquatic environments as they are cosmopolitan in distribution and have been found in every continent although for years MTB were thought to be restricted to habitats with pH values near neutral and at ambient temperature. Recently, however, moderate thermophilic and alkaliphilic MTB have been described including: an uncultured, moderately thermophilic magnetotactic bacterium present in hot springs in northern Nevada with a probable upper growth limit of about 63 °C; and several strains of obligately alkaliphilic MTB isolated in pure culture from different aquatic habitats in California, including the hypersaline, extremely alkaline Mono Lake, with an optimal growth pH of >9.0.

## 1. Introduction

A number of eukaryotic organisms are known to use the Earth’s magnetic field for orientation and navigation (e.g., pigeon, salmon, bats, *etc.*), a behavior referred to as magnetoreception [1]. Although the mechanisms involved in magnetoreception are not well understood, the presence of magnetic mineral crystals has been established in some of these organisms [1]. The most well understood example of magnetoreception is found within the prokaryotes and involves a group of motile, aquatic bacteria known as the magnetotactic bacteria (MTB). In MTB, the magnetoreceptive behavior is called magnetotaxis and is a result of the cells’ ability to biomineralize intracellular, membrane-bounded, tens-of-nanometer-sized crystals of a magnetic mineral consisting of either magnetite (Fe_3_O_4_) or greigite (Fe_3_S_4_) [2]. These structures, termed magnetosomes, cause cells to align along the Earth’s geomagnetic field lines as they swim: the definition of magnetotaxis [3]. MTB are known to mainly inhabit the oxic–anoxic interface (OAI) of aquatic habitats [2] and it is currently thought that the magnetosomes function as a means of making chemotaxis more efficient in locating and maintaining an optimal position for growth and survival at the OAI [3].

Known cultured and uncultured MTB are phylogenetically associated with the *Alpha*-, *Gamma*- and *Deltaproteobacteria* classes of the phylum *Proteobacteria*, the *Nitrospirae* phylum and the candidate division OP3 of the *Planctomycetes*-*Verrucomicrobia*-*Chlamydiae* (PVC) superphylum [4,5,6]. MTB are ubiquitous in almost all types of aquatic environments [2] and are cosmopolitan in distribution as they have been found on every continent [7]. However, despite their broad phylogenetic diversity and wide geographic distribution, no MTB have been classified as extremophilic until recently because: (1) most known cultured MTB are mesophilic with regard to growth temperature and do not grow much above 30 °C (e.g., *Magnetospirillum* species, *Desulfovibrio magneticus*, *Magnetococcus marinus*, *Magnetospira thiophila* and *Magnetovibrio blakemorei*; [8,9,10,11,12]); (2) virtually all studies on uncultured MTB involved sampling sites that were at 30 °C and below; and (3) cultured MTB grow only at about neutral pH while uncultured MTB had never been found in strongly alkaline or acidic habitats. The purpose of this chapter is to present what is known regarding newly discovered MTB that can be considered extremophilic, moderately extremophilic or tolerant to stressors and also to discuss about the potential existence of MTB in other extreme environments and their potential presence on extraterrestrial bodies such as other planets as was once suggested for Mars [13,14]. It is important to note that the work described in this review is focused upon MTB recovered or isolated directly from environmental samples, rather than those inferred indirectly via the detection of magnetotaxis-specific genes in metagenomes.

## 2. Thermophilic Magnetotactic Bacteria

**Figure 1:**
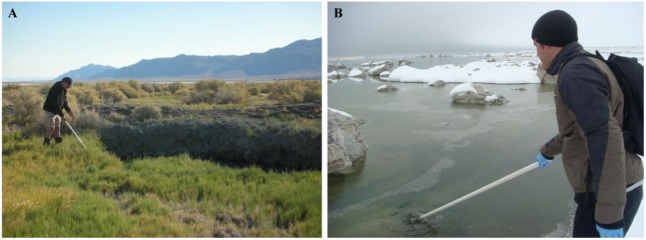
Picture showing the sampling of sediment and water (A) at the Great Boiling Springs (GBS) geothermal field in Gerlach, Nevada, in September 2009. (B) Sampling at Mono Lake, a hypersaline, hyperalkaline endorheic lake situated in California, in February 2010. Tufas, large carbonaceous concretions coming out of the lake can be seen in the background.

In a recent environmental study, Lefèvre *et al.* [15] discovered an uncultured, moderately thermophilic magnetotactic bacterium in hot springs located in northern Nevada. This strain, designated HSMV-1 (*Candidatus* Thermomagnetovibrio paiutensis), was found in mud and water samples collected from the Great Boiling Springs (GBS) geothermal field in Gerlach, Nevada [15] (Figure 1A). GBS is a series of hot springs that range from ambient temperature to ~96 °C [16,17]. The geology, chemistry and microbial ecology of the springs have been described in some detail [16,17].

**Figure 2:**
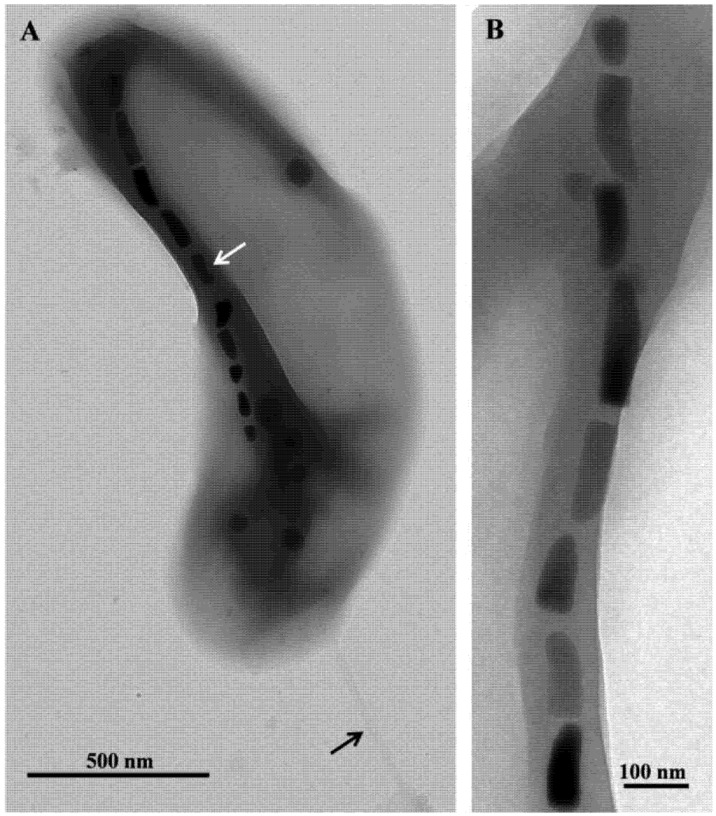
Transmission electron microscope (TEM) images of cells and magnetosomes of the thermophilic magnetotactic bacterium strain HSMV-1 (*Candidatus* Thermomagnetovibrio paiutensis. (A) TEM image of a cell of HSMV-1 showing a single polar flagellum (black arrow) and a single chain of bullet-shaped magnetosomes (white arrow). (B) High magnification TEM image of a magnetosome chain of strain HSMV-1.

Microscopic examination of the samples collected at GBS showed the presence of a single morphotype of MTB in samples taken from nine springs where the temperatures ranged from 32 °C to 63 °C. Cells were small (1.8 ± 0.4 by 0.4 ± 0.1 µm), Gram-negative, vibrioid-to-helicoid in morphology and possessed a single polar flagellum (Figure 2). MTB were not observed in water and mud collected from springs that were 67 °C and higher suggesting the maximum survival and perhaps growth temperature for strain HSMV-1 is about 63 °C. When the water temperature in these pools was <30 °C, cells of strain HSMV-1 were not observed although several other types of MTB were present including greigite-producing, rod-shaped bacteria [15]. Cells of HSMV-1 biomineralize a single chain of bullet-shaped magnetite magnetosomes that traverse the cells along their long axis (Figure 2).

The 16S rRNA gene sequence of strain HSMV-1 places the organism phylogenetically in the phylum *Nitrospirae* with its closest relative in culture being *Thermodesulfovibrio hydrogeniphilus* [18], a non-magnetotactic, thermophilic sulfate-reducing bacterium isolated from a terrestrial Tunisian hot spring with an optimal growth temperature of 65 °C.

Nash [19] also reported the presence of thermophilic MTB in microbial mats at about 45–55 °C adjacent to the main flow in Little Hot Creek and in other springs up to 58 °C all on the east side of the Sierras in California. Cells biomineralized bullet-shaped crystals of magnetite and were phylogenetically affiliated with the phylum *Nitrospirae*. Few additional details were provided [19]. It thus seems likely that these moderately thermophilic MTB are not confined to the GBS and are present in hot springs around the world depending on temperature. Moreover, these studies clearly show that some MTB can be considered at least moderately thermophilic and extend the upper temperature limit for environments where MTB exist and grow and where magnetosome magnetite is deposited.

## 3. Alkaliphilic Magnetotactic Bacteria

While MTB have never been associated with either strongly alkaline or acidic habitats, three strains of obligately alkaliphilic, anaerobic, sulfate-reducing, MTB belonging to the *Deltaproteobacteria* class of the *Proteobacteria*, were recently described [20]. These new magnetotactic strains, designated ML-1, ZZ-1 and AV-1, were isolated in pure culture from three different highly alkaline environments in California, USA. Each has an optimal growth pH of 9.0–9.5.

Strain ML-1 was isolated from the hypersaline, hyperalkaline Mono Lake which is a well-characterized lake located on the arid eastern side of the Sierra Nevada Mountains in California (Figure 1B). The waters of this endorheic, monomictic basin display high alkalinity (pH 9.2 to 10) and salinity (75 g/L to 90 g/L) [21]. Weathering of the surrounding volcanic rocks and hydrothermal inflow results in high sodium and carbonate concentrations [21,22]. Microbial sulfate reduction accounts for 41% of the mineralization of annual primary production in Mono Lake [23]. The pH and salinity of the sample taken from Mono Lake from which strain ML-1 was isolated was 9.8 and 68–70 ppt (parts per thousand), respectively. Strain ZZ-1 was isolated from Soda Spring, a small alkaline spring situated at the Desert Studies Center, a field station of the California State University system, located at the end of Zzyzx Road south of Interstate 15 in California. The salinity at this location was ~27 ppt and the pH 9.5. The third site, where strain AV-1 was isolated from, is an unnamed small pond in Armagosa Valley situated at Death Valley Junction near the border of Nevada and California. This seasonal alkaline pond probably results from underground water flowing through the alkaline desert uplands of Ash Meadows National Wildlife Refuge [24,25] which is in close proximity. This pond is brackish with a salinity of 3 ppt and had a pH of 9.5 at the time of sampling.

**Figure 3:**
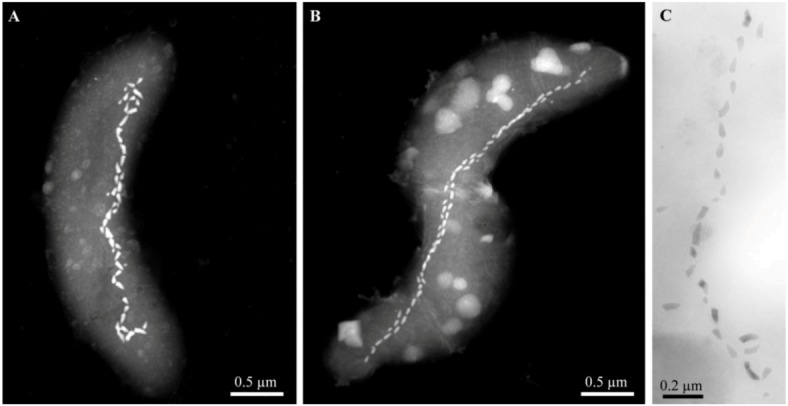
Scanning-transmission electron microscope (STEM) and TEM images of alkaliphilic magnetotactic bacteria. STEM images of cells from (A) the hypersaline Mono Lake, California and (B) a brackish pool at Death Valley Junction (Micrographs courtesy of Tanya Prozorov Ames Laboratory, U.S. Department of Energy, Ames, USA). (C) TEM image of a bullet-shaped magnetosome chain of strain ZZ-1 (Reprinted, with permission, from [20]).

After sampling, the mud and water collected from these three highly alkaline sites contained a significant population (>10^3^ cells/mL) of MTB of a single, morphological type based on light microscopic observations. Cells were helical, possessed a single polar flagellum and contained one or two parallel chains of bullet-shaped, magnetite-containing magnetosomes (Figure 3). The 16S rRNA genes of magnetically-purified, uncultured cells from Mono Lake and the brackish pool at Death Valley Junction were amplified using the polymerase chain reaction and sequenced, and showed that these organisms were closely related (16S rRNA gene sequence identities ≥98.9%) to the non-magnetotactic, alkaliphilic, sulfate-reducing deltaproteobacterium, *Desulfonatronum thiodismutans*, originally isolated from Mono Lake [26]. Magnetically-purified cells were used as inocula in a growth medium for the enrichment of anaerobic, alkaliphilic, sulfate-reducing bacteria modified from Pikuta *et al.* [26] to reflect the salinities of the sampling sites. Cells with the same morphology as those found in the mud and water samples from all sites grew in this growth media. All strains reduced sulfate and used formate and hydrogen as electron donors and were capable of chemolithoautotrophic growth with hydrogen as the electron donor with bicarbonate as the sole carbon source.

The presence of MTB, based on microscopic observations of cells displaying magnetotaxis, has been previously reported from highly alkaline habitats, for example, in Lonar Lake in Maharashtra, India [27,28]. This crater lake [29] is thought to be formed as a result of a meteoritic impact about 50,000 years ago and is a closed basin lake characterized by high alkalinity and salinity. However, no evidence of the presence of magnetosomes and no phylogenetic data were presented for the MTB in Lonar Lake. Further studies are clearly warranted to determine the geographic distribution of alkaliphilic MTB.

Alkaliphilic bacteria must cope with a number of potentially physiological problems living at high pH which includes maintaining their intracellular pH close to neutrality living in an environment where the external pH is >9.0. These organisms have developed a number of interesting metabolic processes to overcome these problems (e.g., higher cytoplasmic buffering capacities) [30]. In the specific case of alkaliphilic MTB, how these prokaryotes synthesize large numbers of magnetosomes in natural highly alkaline environments is particularly interesting in that iron is extremely insoluble at high pH [31]. Therefore, MTB must possess highly efficient mechanisms of iron uptake under these conditions. The fact that strains ML-1, ZZ-1 and AV-1 exist in the reducing anoxic zone in these environments where iron is more likely to be in the more soluble ferrous form, rather than the oxic zone of their habitat probably obviates part of this problem.

## 4. Potential for the Presence of MTB in Other Extreme Environments

In general, MTB are gradient-loving (e.g., oxygen and/or sulfide concentration gradients), microaerophilic or anaerobic microorganisms that are primarily located at or just below the OAI in water columns and sediments of chemically-stratified aquatic environments [2]. In theory, all chemically-stratified, aquatic environments having gradients with the appropriate physical-chemical conditions such as a suitable redox potential and the presence of enough soluble iron, could contain MTB. Thus there is no known reason why extremophilic including acidophilic, piezophilic, halophilic or psychrophilic bacteria could not have acquired the ability to biomineralize magnetosomes during their evolution. In fact, it is possible that these organisms have even been isolated in the past without the realization that the isolated strains were magnetotactic. For example, in an earlier section, we described three strains of alkaliphilic, sulfate-reducing MTB as strains of *Desulfonatronum thiodismutans* based on 16S rRNA gene sequences and phenotypic characteristics. This finding raises the question whether the type species of *D. thiodismuans* was magnetotactic when first isolated from the environment but lost the magnetotactic trait during continued cultivation [20]. This is certainly possible as many cultivated MTB are known to lose this trait relatively easily in culture [32,33,34], sometimes from the loss of a magnetosome genomic island in which the genes for magnetite biomineralization are located [32,34]. In turn, a larger, perhaps more interesting and important question is whether and how many magnetotactic prokaryotic organisms have been characterized from the environment or isolated and deposited in culture collections but have never been recognized as magnetotactic for various reasons. This may be most applicable to the sulfate-reducing bacteria as all cultured dissimilatory sulfate-reducing MTB including *Desulfovibrio magneticus* and the alkaliphic MTB described above, display only a weak magnetotactic response and ability to biomineralize magnetite magnetosomes when grown on sulfate as a terminal electron acceptor [35]. This is most likely due to scavenging of the available iron in the growth medium by sulfide produced during sulfate-reduction.

### 4.1. Piezophilic and Psychrophilic MTB

The deep-sea piezosphere comprises the volume of the oceans at a depth of 1,000 m and greater, with hydrostatic pressures of greater than 100 atmospheres or 10 megapascal (1 atm = 1.013 bar = 0.1 MPa), and accounts for about 75% of the total volume of the world’s oceans [36]. Living MTB have been found in shallow hemipelagic sediments, collected at the depth of ~600 m, from the Santa Barbara Basin in the eastern Pacific with a temperature of 8 °C [37]. No phylogenetic data is available from this study however, only the morphology of the cells and the shape of their magnetosomes were reported. Cells were vibrioid and rod-shaped but the most common observed morphology was coccoid. Magnetosome crystals in these bacteria were composed of magnetite and were cuboidal, elongated prismatic (rectangular in projection) or irregular in shape. The presence of biogenic, ultra-fine-grained, single-domain magnetite (presumably from MTB) in the surface sediments of Santa Barbara Basin was also reported. These magnetite crystals were typical of MTB in their morphologies which included cuboidal, elongated prismatic and teardrop- and bullet-shaped. This study was the first to describe MTB in deep sediments and it was suggested that MTB are the source of the primary remanence carrier in marine sediment.

Petermann and Bleil [38] later reported the presence of MTB in pelagic and hemipelagic sediments of the eastern South Atlantic Ocean. In this study, MTB of different morphologies (cocci, spirilla, vibrioid and rod-shaped) were found at water depths to about 3,000 m in the African continental margin (off Namibia, between the equator and 30 °S) and on the Walvis Ridge in a pelagic environment (about 1,400 km off the coast) situated on a seamount at a water depth of 1,007 m. They showed that the number of MTB in sediment core samples stored at ~25 °C for 12 h significantly declined and those that survived showed less swimming behavior. In samples stored at ~2 °C, MTB could still be detected after several months. It was concluded that at least some marine MTB might be at least facultatively psychrophilic. To test for piezophilic potential in marine MTB, samples of intertidal sediments of the North Sea containing MTB were brought to a water depth of 3,100 m for 24 h [38]. No decrease in numbers or swimming activity of individual cells was detected after recovery indicating that these bacteria are somewhat piezotolerant. 

Living MTB were recently discovered in lakes in the Antarctic [39] clearly demonstrating the existence of at least facultatively psychrophilic MTB. In sum, these studies suggest that the possibility of the existence of obligate piezophilic and psychrophilic bacteria cannot be dismissed.

### 4.2. Acidophilic MTB

Acidophiles are defined as those bacteria having an optimal growth pH of ≤3 [40]. To our knowledge, there are no reports of MTB in highly acidic environments such as acid mine drainage or bogs. The formation of magnetite under these conditions might pose a problem for prokaryotes as magnetite produced through chemical means or through biologically induced biomineralization [41] by non-magnetotactic bacteria (e.g., dissimilatory iron-reducing bacteria) does not appear to be thermodynamically favored at very low pH [42]. However, despite living at very low pH, acidophilic bacteria must maintain an intracellular pH of near neutrality [40] and thus it seems like it may be possible for certain acidophilic species to be capable of biomineralizing intracellular magnetosomes. The fact that iron is more soluble and thus likely more bioavailable under acidic conditions suggests that soluble iron in such environments should not be a limiting factor in magnetosome biomineralization by MTB.

### 4.3. Halophilic Magnetotactic Bacteria

There are no reports specific to halophilic MTB; however there are several studies describing the existence of MTB in hypersaline environments (having salinity levels greater than that of seawater ~35 ppt). Multicellular magnetotactic prokaryotes (MMP) such as *Candidatus* Magnetoglobus multicellularis thrive in the hypersaline Araruama lagoon which has a maximum salinity of 60 ppt [43]. MMPs and other types of MTB are also present in the hypersaline Salton Sea (salinity ~50 ppt) [6,44]. The site from where the sample containing MTB at the Salton Sea was collected was a reddish pool containing large numbers of halophilic prokaryotes as determined by microscopic observation. One species of MTB, designated strain SS-5, from this pool was isolated in pure culture but this strain does not appear to require high concentration of salt to grow. The site from which the alkaliphilic magnetotactic strain ML-1 was isolated in Mono Lake had a salinity of 68 ppt and thus increased the upper limit of salinity where MTB have been found [20]. However like strain SS-5, high salinity is not required for the growth of strain ML-1. Thus some MTB can be considered moderately halophilic and it is possible that halophilic MTB exist as some halophilic strains are known to have a similar metabolism to known MTB (e.g., dissimilatory sulfate reduction) [45] and to be phylogenetically related to MTB (e.g., strain SS-5 related to *Thiohalocapsa marina* [6]).

### 4.4. Magnetotactic Bacteria as Putative Biosignatures for Extraterrestrial Life

MTB have had a major impact on the field of astrobiology. Magnetite crystals morphologically similar to those present in some magnetosomes of MTB living in the present have been found in the Martian meteorite ALH84001 [14,46,47,48,49]. These crystals, putative remains of MTB, have been referred to as “magnetofossils” and have been used as evidence for the past presence of MTB in the meteorite ALH84001 as well as in ancient sediments on Earth dated to about 2 billion years ago [50]. The presence and interpretation of these crystals in Martian meteorite ALH84001 have evoked great controversy and debate particularly because magnetite crystals similar to the ones found in this meteorite can be synthesized in the laboratory without the presence of microorganisms [51,52]. If the magnetite crystals in this meteorite are indeed biogenic, the implication is that bacterial life had existed on ancient Mars [13,14,46,47,48,49,51,52,53]. In turn, this debate has led to a number of criteria to be used to distinguish biogenic magnetite from inorganically-produced magnetite [31,46,54,55,56,57].The discovery and isolation of obligately alkaliphilic MTB is discussed in a previous section and clearly demonstrates that some magnetotactic species can be considered extremophilic. Because MTB had never been considered to inhabit extreme environments, highly alkaline habitats have apparently not been searched for magnetofossils. Chemical analyses of soil samples of Mars indicate a period of highly alkaline conditions on the planet in the past [58,59,60]. Moreover equilibrium modeling based on measured Ca^2+^ and Mg^2+^ concentrations were consistent with carbonate equilibrium for a saturated solution [59,60] and thus carbonate buffering appears to be significant in some Martian soils as it is in Mono Lake. Mono Lake has been used by researchers at a number of institutions, including the National Aeronautics and Space Administration (NASA), as a model for extreme environments that might be comparable to those on the planet Mars [61]. It would be interesting to determine whether bullet-shaped magnetite crystals like those in strains ML-1 are incorporated and preserved as magnetofossils in carbonate minerals, such as the unusual carbonate structures known as tufas, abundant in Mono Lake, as they appear to do in sedimentary carbonates in marine environments [62,63] and in carbonates in the Martian meteorite ALH84001 [47]. Between 2018 and 2023, a mission is planned by NASA to collect rock and dust samples from Mars and to return them to Earth for analysis. It will be interesting and intriguing if magnetofossils are found in these samples.

## 5. Conclusions

Almost 50 years after the initial discovery of MTB by Salvatore Bellini in 1963 [64,65] and 40 years after the rediscovery and formal publication of these organisms by Richard P. Blakemore [66], it is only recently that extremophilic MTB have been observed and described. These findings clearly raise the possibility that magnetotactic microorganisms might exist in other extreme environments that have never been sampled and examined for their presence. Do MTB exist in environments characterized by very high pressure, by extreme cold, highly acidic or highly saline? To address this question, more sampling missions in extreme environments will need to be done by researchers using specific culture- and non-culture-based techniques. However, based on the recent results described in this chapter, the potential of finding MTB in other extreme environments is high. Moreover, considering the relatively small number of groups that study these intriguing organisms, it seems that the known ecological diversity of MTB is seriously underestimated.
